# Disparities in pulmonary fibrosis care in the United States: an analysis from the Nationwide Inpatient Sample

**DOI:** 10.1186/s12913-018-3407-0

**Published:** 2018-08-08

**Authors:** Adam W. Gaffney, Steffie Woolhander, David Himmelstein, Danny McCormick

**Affiliations:** 1 Division of Pulmonary and Critical Care Medicine, Cambridge Health Alliance, 1493 Cambridge Street, Cambridge, MA 02138 USA; 2 Harvard Medical School, Boston, MA USA; 3 City University of New York at Hunter College, New York, NY USA; 4 Department of Medicine, Cambridge Health Alliance, Cambridge, MA USA

**Keywords:** Disparities, Idiopathic pulmonary fibrosis, Lung transplantation

## Abstract

**Background:**

Idiopathic pulmonary fibrosis is a disease with high morbidity and mortality. Care for these patients, including lung transplantation, may provide significant benefits, but is resource-intensive and expensive. Disadvantaged patients with IPF may hence be at risk for receiving inferior care.

**Methods:**

We analyzed data from the Nationwide Inpatient Sample, a database consisting of all hospitalizations from a 20% sample of US hospitals. We identified adults hospitalized with IPF between 1998 and 2011 using ICD-9 codes. We assessed the effect of insurance coverage and socioeconomic status (SES) on lung transplantation, a treatment that may improve survival. We also examined the effect of coverage and SES on mortality, as well as discharge to inpatient rehabilitation and receipt of a lung biopsy, two markers of the intensity of care delivered. We used multiple logistic regression to adjust for patient and hospital characteristics.

**Results:**

We identified 148,877 hospitalizations that met our definition of pulmonary fibrosis. In the main adjusted analyses, hospitalizations of patients with Medicaid (OR 0.30, 95% CI 0.16–0.57) or no insurance (OR 0.22, 95% CI 0.07–0.72) were less likely to result in a lung transplantation compared to hospitalizations of those with non-Medicaid insurance. Those of lower SES were also less likely to undergo transplantation, while hospitalized patients with Medicaid and the uninsured were less likely to be discharged to inpatient rehabilitation or to receive a lung biopsy.

**Conclusions:**

Among hospitalized patients with IPF, those with lower SES, Medicaid coverage and without insurance were less likely to receive several clinical interventions.

**Electronic supplementary material:**

The online version of this article (10.1186/s12913-018-3407-0) contains supplementary material, which is available to authorized users.

## Background

Health disparities affect disadvantaged patients with a range of pulmonary diseases [1–3]. Yet inequalities in care for patients with interstitial lung diseases—inflammatory and fibrotic conditions affecting the alveolar wall, such as idiopathic pulmonary fibrosis (IPF) [4]—have not been well studied.

IPF is a chronic interstitial lung disease with a progressive, debilitating, and eventually deadly course. Its diagnosis requires a surgical lung biopsy in some but not all patients [5]. No cure is available; however, new treatments ameliorate the disease [6, 7]. Lung transplantation may improve survival [8, 9], and current guidelines support its use in appropriate patients with IPF [5]. Pulmonary rehabilitation is also recommended [5], with some evidence pointing to improved quality of life and exercise tolerance for such patients [10]. However, these are expensive, resource-intensive interventions. Hence, disadvantages patients with IPF may be at risk of receiving less care for their illness.

Using a large inpatient administrative database, we investigated disparities by insurance and socioeconomic status (SES) for a range of clinical interventions and outcomes among patients hospitalized in the United States with pulmonary fibrosis.

## Methods

### Study design and patient population

We used data from the Nationwide Inpatient Sample (NIS) (Healthcare Cost and Utilization Project, Agency for Healthcare Research and Quality), an administrative claims database consisting (until 2012) of all hospitalizations drawn from a sample of 20% of US hospitals, and then weighted to be nationally representative of all US hospitalizations [11, 12].

We identified adults aged 18 years or older who were hospitalized between 1998 and 2011 with probable IPF based on diagnostic coding. In the NIS database, hospitalizations receive up to 15 ICD-9 codes. Previous investigations have identified subjects with IPF based on the presence (prior to 2012) of an ICD-9 code for “idiopathic fibrosing alveolitis” (516.3), together with no ICD-9 code for any other interstitial lung disease (ILD) or ILD-related diagnosis (e.g. connective tissue disease) other than the non-specific code for post-inflammatory fibrosis (515) (see Additional file 1) [13–15]. This is likely a conservative approach, since some subjects with IPF may be coded with 515 (post-inflammatory fibrosis) and not 516.3 [14]. Thus, for our main study analysis, we followed Raghu et al. in excluding patients with diagnostic codes for other ILDs or ILD-related diagnoses [13–15], yet followed others [16, 17] in including subjects with either code 516.3 or 515. Because the ICD-9 coding for IPF changed in 2012, for consistency, we only included hospitalizations from the years 1998–2011.

Our main analysis included all hospitalizations with either code 516.3 or 515 as one of the first two diagnoses, and without any secondary codes (see Additional file 1). In a sensitivity analysis, we created a more narrowly defined cohort consisting of hospitalizations with the more specific code 516.3 as one of the top two diagnostic codes, and without any secondary code apart from 515.

### Data and measurements

We assessed the effects of insurance status on treatment, based on the 6-category variable “expected primary payer”, which we simplified into three-categories: (1) “non-Medicaid insurance” (Medicare, private insurance, or “other insurance”), (2) Medicaid, and (3) uninsured (“self-pay” or “no charge”). Medicare is a universal federal public health insurance program that primarily covers essentially all adults aged 65 and older, although younger individuals who are disabled or who have end-stage kidney disease are also eligible. Medicaid is a means-tested federal-state public health insurance program covering those of low-income, who traditionally also belonged to certain groups (e.g., dependent children and their parents, the disabled, and pregnant women).

In a sensitivity analysis, we excluded subjects aged 65 or older as well as younger patients with Medicare, and recoded insurance status into a three-category variable: (1) private insurance (“private insurance” or “other”), (2) Medicaid, and (3) uninsured (“self-pay” or “no charge”).

We also assessed the effects of SES on treatments received. We defined SES by the median income of the ZIP Code in which the patient resided using the variable ZIPINC_QRTZ. This variable was categorized into quartiles with quartile 1 representing the lowest and quartile 4 representing the highest income levels. ZIP Code income data were updated annually for years 2003 and beyond. However, for the years 1998–2002, a somewhat different 4-category variable was used for the median income of the patient’s ZIP Code, and which is based on 1999 demographics [18].

Our first outcome was lung transplantation during the hospitalization, defined by having one or more procedure codes for lung transplant (33.5, 33.50, 33.51, or 33.52). The second outcome was “death without transplant” among non-transplanted patients.

The third outcome was discharge to a rehabilitation facility For this, we generated a binary variable to indicate transfer to a rehabilitation facility vs. any other disposition using the NIS variables “Dispub92” (for pre-2008 discharges, which follows the UB-92 claim form) or “Dispub04” (for discharges in 2007–2011, which follows the UB-04 claim form). (The UB-92 and UB-04 are billing forms submitted to insurers by healthcare facilities for reimbursement). Hospitalizations with code 62 were considered to be discharged to a rehabilitation facility (see Additional file 1, note). Although patients discharged to a skilled nursing facility (a separate code) sometimes also receive rehabilitative services following discharge, we opted to use the more specific discharge disposition (i.e. code 62) as it likely reflects a higher intensity of rehabilitative care [19]. Because no patients had a discharge disposition of code 62 earlier than 2001, earlier years were excluded for the rehabilitation analysis, as were patients who died, had missing death data, or were transplanted during the hospitalization.

Our fourth outcome was receipt of a thorascopic lung biopsy (procedure code 33.20). This code did not exist until 2007. Hence, earlier years were excluded from this analysis. We do not assume that thorascopic lung biopsy or inpatient rehabilitation are independent metrics of quality. Rather, we view them as markers for the overall intensity of care delivered, analogous to the approach of Lyon et al. who investigated disparities in procedures received by critically-ill patients [20].

We included individual and hospital-level characteristics in our models because of their plausible role as confounders. Individual characteristics included gender, age (on admission, treated as a continuous variable), race (white, black, Hispanic, Asian or Pacific Islander, Native American, or other), and year of discharge (treated as a categorical variable). Comorbidities were coded using the Healthcare Cost and Utilization Project (HCUP) Elixhauser Comorbidity Software, which generates 29 binary comorbidity variables based on diagnostic codes [21]. We then used HCUP software to generate the Agency for Healthcare Research and Quality (AHRQ) Elixhauser Comorbidity index for in-hospital mortality for each hospitalization [21, 22], which we included in our models as a continuous variable to adjust for disease severity.

Models also included hospital characteristics, including: census region (Northeast, Midwest, South, and West), hospital location (rural vs. urban), hospital teaching status (nonteaching vs. teaching), and hospital bed-size by bed number (small, medium, or large). Hospitalizations with missing data on exposure or covariates were excluded.

### Analysis plan

Baseline characteristics were determined using PROC SURVEYFREQ or PROC SURVEYMEANS using the SAS statistical software program (Cary, N.C). For each of the four outcomes, we performed multivariate logistic regression using the SAS procedure PROC SURVEYLOGISTIC, which accounts for the complex sample design. All analyses were appropriately weighted using weights supplied by the NIS to produce nationally representative estimates [23]. Year of hospitalization was used as a stratification variable. Standard errors were adjusted for clustering by discharge hospital using the clustering and sampling variables provided by the NIS. Because analyses were performed on subsets of the NIS, we followed the steps outlined by HCUP documentation (Additional file 1) [24] to include all hospitals and ensure appropriate estimation of variances.

Because no lung transplantations occurred at non-teaching or non-urban hospitals we excluded these variables from analyses of lung transplantation. Notably, the validity of the model fit was questionable for the analysis of rehabilitation in both sensitivity analyses, and for VATS biopsy in the sensitivity analysis that used the more narrow IPF definition.

This study received “exempt” status from the Institutional Review Board of the Cambridge Health Alliance.

## Results

**Fig. 1:**
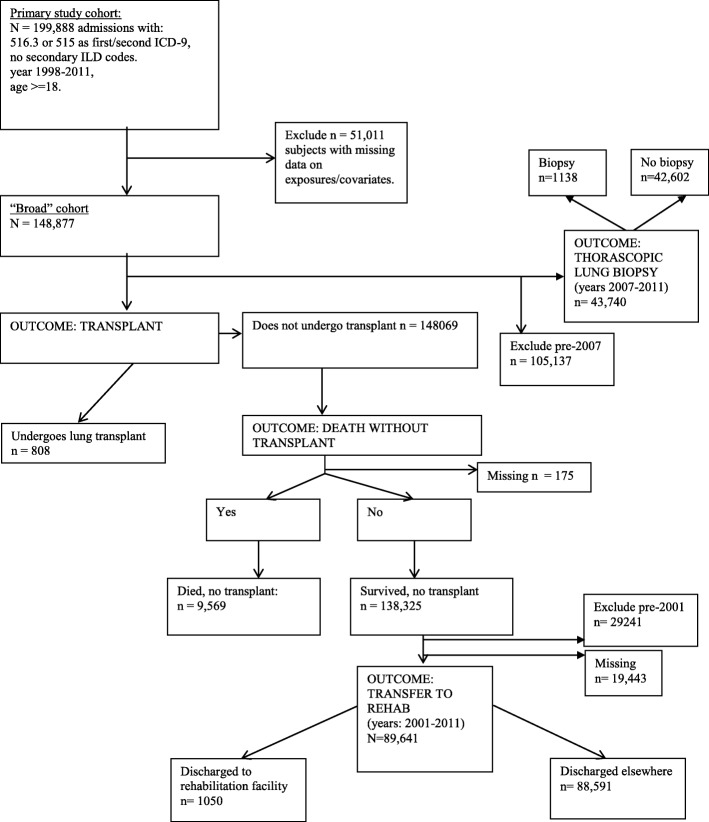
Flow chart of main study population formation with outcomes

Figure 1 outlines the cohorts used in our main analyses for each of the four outcomes measures. There were 199,888 hospitalizations with codes 516.3 or 515 as the first or second diagnostic code for the years 1998–2011 among adults aged > = 18. After excluding 51,011 hospitalizations that were missing data on any predictor variable or covariate, 148,877 hospitalizations remained. The more narrow definition of IPF, used for our sensitivity analysis, produced a much smaller cohort (17,783 hospitalizations). The cohort of those under age 65 without Medicare was also substantially smaller (34,629 hospitalizations).

In our main cohort, an unweighted total of 808 patients (0.5% of 148,877) received a lung transplant, while 9569 died (6.5% of the 148,069 not transplanted), 1050 were transferred to a rehabilitation facility (1.2% of the 89,641 eligible survivors with data on disposition who were not transplanted), and 1138 underwent a thorascopic lung biopsy (2.6% of the 43,740 eligible).

Table 1 displays the weighted characteristics of the main cohort according to insurance status. The mean age was 70.5 years and 55.7% were female. Patients with Medicaid or no insurance were younger than other patients, were more likely to be a racial/ethnic minority and live in lower income zip codes.

**Table 1:** Characteristics of patients hospitalized with pulmonary fibrosis stratified by insurance status^a^

	Total	Non-Medicaid insurance (91.6%)	Medicaid insurance (5.7%)	Uninsured (2.1%)
Age (years)	70.5	71.9	54.6	53.8
Female	55.7%	55.3%	62.8%	50.4%
Race				
White	78.2%	81.0%	42.7%	52.2%
Black	8.7%	7.6%	22.3%	19.7%
Hispanic	8.8%	7.5%	24.6%	19.7%
Asian or Pacific	1.9%	1.7%	4.8%	2.4%
Islander				
Native American	0.4%	0.4%	0.9%	0.6%
Other	2.1%	1.9%	4.6%	5.4%
Income Quartile of Zipcode				
1st	27.3%	26.1%	43.6%	37.5%
2nd	26.4%	26.4%	26.8%	28.9%
3rd	24.2%	24.6%	18.7%	20.9%
4th	22.1%	23.0%	10.9%	12.6%
Hospital Region				
Northeast	22.5%	22.5%	22.9%	18.3%
Midwest	17.0%	17.4%	12.1%	12.4%
South	42.0%	42.0%	37.3%	57.4%
West	18.5%	18.1%	27.7%	11.9%
Hospital Location				
Rural	13.7%	14.0%	10.0%	11.8%
Urban	86.3%	86.0%	90.0%	88.2%
Hospital Teach				
Nonteaching	59.1%	60.0%	48.5%	48.9%
Teaching	40.9%	40.0%	51.5%	51.1%
Bedsize of hospital				
Small	12.3%	12.5%	10.1%	12.7%
Medium	26.0%	25.9%	26.7%	25.8%
Large	61.7%	61.6%	63.2%	61.5%
AHRQ Elixhauser Comorbidity Index	4.6	4.7	3.7	2.6

^a^Nationwide estimates based on weights supplied by NIS

In the fully adjusted models (Table 2), patients with Medicaid were less likely (OR 0.30, 95% CI 0.16, 0.57) to undergo a lung transplant than those with non-Medicaid insurance, as were those with no insurance (OR 0.22, 95% CI 0.07, 0.72). Patients in the lower three quartiles of median ZIP Code income were less likely to receive a transplant than patients in the highest quartile (OR for lowest vs. highest income quartile, 0.46, 95% CI 0.32, 0.66).

**Table 2:** Adjusted odds of four outcomes in IPF patients (ICD9 516.3 or 515)^a^

	Lung Transplant		Death		Rehabilitation Transfer		VATS Biopsy	
	Odds Ratio (95% CI)	*P*-Value	Odds Ratio (95% CI)	P-Value	Odds Ratio (95% CI)	P-Value	Odds Ratio (95% CI)	P-Value
Insurance								
Non-medicaid	*Reference*		*Reference*		*Reference*		*Reference*	
Medicaid	0.30 (0.16, 0.57)	< 0.001	1.00 (0.89, 1.12)	0.95	0.53 (0.33, 0.85)	0.01	0.46 (0.35, 0.60)	< 0.001
Uninsured	0.22 (0.07, 0.72)	0.01	1.12 (0.92, 1.35)	0.26	0.41 (0.18, 0.93)	0.03	0.52 (0.35, 0.77)	< 0.01
ZIP Income Quartile								
Quartile 1	0.46 (0.32, 0.66)	< 0.001	0.93 (0.87, 1.00)	0.04	1.16 (0.92, 1.45)	0.21	1.01 (0.81, 1.25)	0.96
Quartile 2	0.56 (0.43, 0.73)	< 0.001	0.91 (0.85, 0.97)	< 0.01	1.00 (0.80, 1.24)	0.97	1.09 (0.90, 1.33)	0.38
Quartile 3	0.71 (0.57, 0.89)	< 0.01	0.96 (0.90, 1.02)	0.19	1.11 (0.90, 1.36)	0.33	0.98 (0.81, 1.18)	0.81
Quartile 4 (Highest)	*Reference*		*Reference*		*Reference*		*Reference*	

^a^Analyses of death, rehab, and VATS were adjusted for age, race, gender, insurance, year, zip income quartile, hospital region, hospital location, hospital teaching status, hospital bedsize, and AHRQ Elixhauser Comorbidity index for in-hospital mortality. Analyses of lung transplantation were adjusted for these same variables, but not hospital teaching status nor urban/rural location, because the proportion of lung transplantations exhibiting these characteristics was either 0% or 100%

Patients with Medicaid (OR 0.53, 95% CI 0.33, 0.85) or no insurance (OR 0.41, 95% 0.18, 0.93) were less likely to be discharged to a rehabilitation facility. Patients with Medicaid were also less likely to undergo a VATS lung biopsy (OR 0.46, 95% CI 0.35, 0.60), as were those with no insurance (OR 0.52, 95% CI 0.35, 0.77).

There was no relationship between insurance status and in-hospital death. Those in ZIP Code income quartile 1 (OR 0.93, 95% CI 0.87, 1.00) and quartile 2 (OR 0.91, 95% CI 0.85, 0.97), but not those in quartile 3, had a reduced odds of death as compared to quartile 4.

The results of analyses confined to those aged less than 65 and without Medicare are shown in Table 3. Those with Medicaid (OR 0.28, 95% CI 0.15, 0.51) and the uninsured (OR 0.20, 95% CI 0.06, 0.63) continued to have significantly lower odds of lung transplant, as well as reduced odds of a VATS biopsy. Those in ZIP Code income quartiles 1 and 2, but not quartile 3, continued to have significantly reduced odds of lung transplantation. However, the association between insurance and rehabilitation transfer was no longer significant, although the model for this outcome demonstrated questionable fit. There were no associations between insurance status or zip income quartile and death.

**Table 3:** Sensitivity Analysis: Adjusted odds of four outcomes among persons age < 65 (ICD9 516.3 or 515)^a^

	Lung Transplant		Death		Rehabilitation Transfer^b^		VATS Biopsy	
	Odds Ratio (95% CI)	P-Value	Odds Ratio (95% CI)	P-Value	Odds Ratio (95% CI)	P-Value	Odds Ratio (95% CI)	P-Value
Insurance								
Non-medicaid	*Reference*		*Reference*		*Reference*		*Reference*	
Medicaid	0.28 (0.15, 0.51)	< 0.001	0.96 (0.82, 1.12)	0.57	0.78 (0.44, 1.38)	0.39	0.43 (0.32, 0.56)	< 0.001
Uninsured	0.20 (0.06, 0.63)	0.01	0.84 (0.66, 1.08)	0.18	0.45 (0.16, 1.25)	0.12	0.50 (0.34, 0.75)	< 0.01
ZIP Income Quartile								
Quartile 1	0.50 (0.34, 0.72)	< 0.001	0.96 (0.81, 1.13)	0.60	1.04 (0.53, 2.04)	0.91	1.29 (0.97, 1.73)	0.08
Quartile 2	0.63 (0.47, 0.84)	<.01	0.87 (0.74, 1.02)	0.08	1.30 (0.73, 2.31)	0.38	1.14 (0.88, 1.49)	0.32
Quartile 3	0.81 (0.64, 1.03)	0.09	0.96 (0.82, 1.12)	0.57	1.24 (0.69, 2.22)	0.47	1.05 (0.81, 1.36)	0.74
Quartile 4 (Highest)	*Reference*		*Reference*		*Reference*		*Reference*	

^a^Analyses of death, rehab, and VATS were adjusted for age, race, gender, insurance, year, zip income quartile, hospital region, hospital location, hospital teaching status, hospital bedsize, and AHRQ Elixhauser Comorbidity index for in-hospital mortality. Analyses of lung transplantation were adjusted for these same variables, but not hospital teaching status and urban/rural location, because the proportion of lung transplantations exhibiting these characteristics was either 0% or 100%

^b^The analysis for rehabilitation transfer demonstrated questionable model fit

In the sensitivity analysis using the narrower definition of IPF (hospitalizations with ICD-9 code 516.3), a reduced odds of transplantation (OR 0.24, 95% CI 0.11, 0.53) was again seen for those with Medicaid, with a nonsignificant trend towards a lower likelihood of transplantation amongst the uninsured (Additional file 2: Table S1). Those in the lower three income quartiles continued to have a significantly lower odds of transplant as well (quartile 1 v. quartile 4 OR 0.46, 95% CI 0.29, 0.75). No associations were seen between insurance status or SES and death, VATS biopsy, or rehabilitation transfer, although the latter two models had questionable fit.

## Discussion

Among patients hospitalized with pulmonary fibrosis in the US, those with Medicaid (a public insurance program for those of low income), the uninsured, and patients of lower SES were much less likely to receive a lung transplant. Those with Medicaid and the uninsured were also less likely to be discharged to a rehabilitation facility or undergo a lung biopsy.

Healthcare inequalities in patients with pulmonary disease [2] likely arise from several sources. Low SES is itself associated with lower pulmonary function, which may stem from a variety of exposures [25–27]. However, unequal healthcare access may also be a factor. Uninsured patients with lung cancer are less likely to receive potentially curative therapy [3], while those with cystic fibrosis die at a younger age [28]. People with asthma who are uninsured, poor, black, or Hispanic have inferior healthcare access and worse outcomes [1, 29, 30]. Children with Medicaid are more likely than privately insured children to be denied an appointment to see a specialist—including pulmonary specialists [31]. And among adults with cystic fibrosis, Medicaid coverage and low SES are independently associated with reduced odds of being accepted onto a lung transplant waiting list [32]. Non-population based studies also suggest that racial disparities may impact patients with IPF, including in the transplantation process [33–36].

For other organs, evidence suggests racial disparities throughout the transplantation process [37–39]. For instance, among individuals on a liver transplant waiting list, Hispanics are less likely to go on to receive an organ [37]. And with respect to insurance-related disparities, a study using NIS data found that whereas organ *donors* are more likely to be uninsured than other hospitalized patients, organ *recipients* were virtually always insured [40].

Our study has some limitations. While the NIS provided a very large, nationally representative sample—which allows population-based assessment of rare events like transplantation—it represents hospitalizations, not individual patients. Thus, a factor that led to increased hospitalizations among disadvantaged IPF patients could, by increasing the denominator (i.e. the number of admissions per patient), confound our results. However, given the very low ORs of transplantation among the uninsured and those with Medicaid, any such confounder would have to increase total hospitalizations several-fold to explain the observed disparities. Moreover, evidence suggests that the uninsured are actually less likely to be hospitalized than the insured [41], which would tend to bias our results towards the null, at least for the uninsured.

Our findings do not necessarily demonstrate “within-hospital” disparities. The disparities we observed could also result from the fact that, as compared to more disadvantaged patients, advantaged patients may be more likely to be admitted (or transferred) to hospitals that provide a higher intensity of care (i.e. “between hospital” disparities). However, this was our outcome of interest, since disadvantaged patients with IPF may not be referred to lung transplantation centers to begin with.

Another limitation was our measure of SES, which was based on ZIP code median income. Area-level median income describes only one component of SES. Family income, wealth, and education are other measures of SES that could potentially have different associations with our outcomes of interest, although this was not something we could evaluate in this study.

Since we lacked patient level data on the severity of IPF, another potential concern is that the lower observed rates of lung transplantation for disadvantaged patients reflect a lower severity of pulmonary illness in these groups. While we controlled for age and Elixhauser comorbidities, the possibility of residual confounding by pulmonary disease severity persists. However, there is no clear biological rationale why disadvantaged patients should have less severe lung disease. Moreover, if they did, we would expect lower in-hospital mortality, which was overall not the case (the slightly reduced odds ratios for death seen for two of the zip income quartiles were not observed in sensitivity analyses).

We also found that those with Medicaid and the uninsured were less likely to be transferred to inpatient pulmonary rehabilitation or to have a VATS biopsy. Pulmonary rehabilitation is clinically beneficial [10] for some patients and is recommended for IPF [5]. Although outpatient rehabilitation is much more typical, differences in transfer to inpatient rehabilitation facilities nonetheless can be seen as a disparity in treatment. Although lung biopsy is not necessary in all patients with interstitial lung disease (and in some instances might reflect overtreatment), lower rates of biopsies suggest that care decisions for hospitalized patients with pulmonary fibrosis may, in part, be based on insurance status rather than clinical need. Finally, the overall absence of consistent disparities in death is not surprising given the lack of evidence demonstrating that any specific therapy improves survival for hospitalized patients with IPF.

What should pulmonologists make of these findings? To some extent, it is not surprising that uninsured patients receive fewer lung transplantations [42]. Transplanting a patient who is unable to obtain immunosuppressive medications following transplant could actually cause more harm than good. However, this does not mean that such disparities are justifiable. Similarly, the reduced odds of transplant among patients with Medicaid insurance—similar to what has been seen in patients with cystic fibrosis [32]—is troubling, particularly given that this program covers almost 75 million Americans [43]. Our findings reinforce the urgency of physician advocacy for health coverage that is not only universal, but equal [44].

There is evidence that “single tier” health insurance systems may indeed be more equitable [45]. For instance, a study of patients referred to kidney transplant clinics in the Veterans Administration Health Care system found no differences by race in the time to acceptance of a kidney transplant [46]. And a recent study comparing patients with cystic fibrosis in the US with those in Canada—which has a single-payer system—found that the Canadians lived a decade longer, and also received more transplants [47].

## Conclusions

We found that in the United States, hospitalized individuals with idiopathic pulmonary fibrosis who had no insurance, public insurance (Medicaid), or who were from lower socioeconomic status areas were less likely to receive several clinical interventions. Clinical pulmonologists should advocate for more equitable access to lung transplant, and all other treatments, both within our institutions and our society [44].

## Additional files

Additional file 1: Diagnostic codes for other ILDs. Lists ICD-9 codes for other interstitial lung diseases. Note on disposition Includes notes on disposition variables. (DOCX 15 kb)

Additional file 2: **Table S1.** Adjusted odds of outcomes, IPF “narrow” cohort (ICD9 516.3 only). Results of the sensitivity analysis using the narrower definition of IPF (hospitalizations with ICD-9 code 516.3 only). (DOCX 19 kb)

## Abbreviations

HCUP: Healthcare Cost and Utilization Project
ILD: Interstitial lung disease
IPF: Idiopathic pulmonary fibrosis
NIS: Nationwide Inpatient Sample
SES: Socioeconomic status
VATS: Video-assisted thorascopic surgery

## References

[1] Ungar WJ, Paterson JM, Gomes T, Bikangaga P, Gold M, Kozyrskyj AL, To T (2011). Relationship of asthma management, socioeconomic status, and medication insurance characteristics to exacerbation frequency in children with asthma. Ann Allergy Asthma Immunol.

[2] Schraufnagel DE, Blasi F, Kraft M, Gaga M, Finn PW, Rabe KF; ATS/ERS Committee on Disparities in Respiratory Health: An official American Thoracic Society/European Respiratory Society policy statement: disparities in respiratory health. Am J Respir Crit Care Med. 2013;188(7):865–71. 10.1164/rccm.201308-1509ST 24083859

[3] Slatore CG, Au DH, Gould MK (2010). An official American Thoracic Society systematic review: insurance status and disparities in lung cancer practices and outcomes. Am J Respir Crit Care Med.

[4] Lederer DJ, Martinez FJ (2018). Idiopathic pulmonary fibrosis. N Engl J Med.

[5] Raghu G, Collard HR, Egan JJ, Martinez FJ, Behr J, Brown KK, Colby TV, Cordier J-F, Flaherty KR, Lasky JA (2011). An official ATS/ERS/JRS/ALAT statement: idiopathic pulmonary fibrosis: evidence-based guidelines for diagnosis and management. Am J Respir Crit Care Med.

[6] Richeldi L, du Bois RM, Raghu G, Azuma A, Brown KK, Costabel U, Cottin V, Flaherty KR, Hansell DM, Inoue Y (2014). Efficacy and safety of Nintedanib in idiopathic pulmonary fibrosis. N Engl J Med.

[7] King TE, Bradford WZ, Castro-Bernardini S, Fagan EA, Glaspole I, Glassberg MK, Gorina E, Hopkins PM, Kardatzke D, Lancaster L (2014). A phase 3 trial of Pirfenidone in patients with idiopathic pulmonary fibrosis. N Engl J Med.

[8] George TJ, Arnaoutakis GJ, Shah AS (2011). Lung transplant in idiopathic pulmonary fibrosis. Arch Surg.

[9] Thabut G, Mal H, Castier Y, Groussard O, Brugiere O, Marrash-Chahla R, Leseche G, Fournier M (2003). Survival benefit of lung transplantation for patients with idiopathic pulmonary fibrosis. J Thorac Cardiovasc Surg.

[10] Huppmann P, Sczepanski B, Boensch M, Winterkamp S, Schonheit-Kenn U, Neurohr C, Behr J, Kenn K (2013). Effects of inpatient pulmonary rehabilitation in patients with interstitial lung disease. Eur Respir J.

[11] Introduction to the HCUP Nationwide Inpatient Sample (NIS) 2011**.** https://www.hcup-us.ahrq.gov/db/nation/nis/NIS_Introduction_2011.pdf.

[12] HCUP (2012). National Inpatient Sample (NIS). Healthcare cost and utilization project (HCUP).

[13] Raghu G, Chen S-Y, Yeh W-S, Maroni B, Li Q, Lee Y-C, Collard HR (2014). Idiopathic pulmonary fibrosis in US Medicare beneficiaries aged 65 years and older: incidence, prevalence, and survival, 2001–11. Lancet Respir Med.

[14] Raghu G, Weycker D, Edelsberg J, Bradford WZ, Oster G (2006). Incidence and prevalence of idiopathic pulmonary fibrosis. Am J Respir Crit Care Med.

[15] Raghu G, Chen SY, Hou Q, Yeh WS, Collard HR (2016). Incidence and prevalence of idiopathic pulmonary fibrosis in US adults 18-64 years old. Eur Respir J.

[16] Mannino DM, Etzel RA, Parrish RG (1996). Pulmonary fibrosis deaths in the United States, 1979-1991. An analysis of multiple-cause mortality data. Am J Respir Crit Care Med.

[17] Olson AL, Swigris JJ, Lezotte DC, Norris JM, Wilson CG, Brown KK (2007). Mortality from pulmonary fibrosis increased in the United States from 1992 to 2003. Am J Respir Crit Care Med.

[18] NIS Description of Data Elements. Healthcare Cost and Utilization Project (HCUP). https://www.hcup-us.ahrq.gov/db/nation/nis/nisdde.jsp.

[19] Skolarus LE, Meurer WJ, Burke JF, Prvu Bettger J, Lisabeth LD (2012). Effect of insurance status on postacute care among working age stroke survivors. Neurology.

[20] Lyon SM, Benson NM, Cooke CR, Iwashyna TJ, Ratcliffe SJ, Kahn JM (2011). The effect of insurance status on mortality and procedural use in critically ill patients. Am J Respir Crit Care Med.

[21] Elixhauser Comorbidity Software, Version 3.7. https://www.hcup-us.ahrq.gov/toolssoftware/comorbidity/comorbidity.jsp#pubs. Accessed July 6, 2017.

[22] Moore BJ, White S, Washington R, Coenen N, Elixhauser A (2017). Identifying increased risk of readmission and in-hospital mortality using hospital administrative data: the AHRQ Elixhauser comorbidity index. Med Care.

[23] Producing National HCUP Estimates. https://www.hcup-us.ahrq.gov/tech_assist/nationalestimates/508_course/508course.jsp. Accessed July 7, 2017.

[24] HCUP Methods Series: Calculating Nationwide Inpatient Sample (NIS) Variances for Data Years 2011 and Earlier. https://www.hcup-us.ahrq.gov/reports/methods/2003_02.jsp#appb. Accessed July 7, 2017.

[25] Hegewald MJ, Crapo RO (2007). Socioeconomic status and lung function. Chest.

[26] Gray LA, Leyland AH, Benzeval M, Watt GC (2013). Explaining the social patterning of lung function in adulthood at different ages: the roles of childhood precursors, health behaviours and environmental factors. J Epidemiol Community Health.

[27] Gaffney AW, Hang JQ, Lee MS, Su L, Zhang FY, Christiani DC (2016). Socioeconomic status is associated with reduced lung function in China: an analysis from a large cross-sectional study in shanghai. BMC Public Health.

[28] Curtis JR, Burke W, Kassner AW, Aitken ML (1997). Absence of health insurance is associated with decreased life expectancy in patients with cystic fibrosis. Am J Respir Crit Care Med.

[29] Wood PR, Smith LA, Romero D, Bradshaw P, Wise PH, Chavkin W (2002). Relationships between welfare status, health insurance status, and health and medical care among children with asthma. Am J Public Health.

[30] Forno E, Celedon JC (2012). Health disparities in asthma. Am J Respir Crit Care Med.

[31] Bisgaier J, Rhodes KV (2011). Auditing access to specialty Care for Children with public insurance. N Engl J Med.

[32] Quon BS, Psoter K, Mayer-Hamblett N, Aitken ML, Li CI, Goss CH (2012). Disparities in access to lung transplantation for patients with cystic fibrosis by socioeconomic status. Am J Respir Crit Care Med.

[33] Lamas DJ, Kawut SM, Bagiella E, Philip N, Arcasoy SM, Lederer DJ (2011). Delayed access and survival in idiopathic pulmonary fibrosis. Am J Respir Crit Care Med.

[34] Lederer DJ, Caplan-Shaw CE, O'Shea MK, Wilt JS, Basner RC, Bartels MN, Sonett JR, Arcasoy SM, Kawut SM (2006). Racial and ethnic disparities in survival in lung transplant candidates with idiopathic pulmonary fibrosis. Am J Transplant.

[35] Lederer DJ, Arcasoy SM, Barr RG, Wilt JS, Bagiella E, D'Ovidio F, Sonett JR, Kawut SM (2006). Racial and ethnic disparities in idiopathic pulmonary fibrosis: a UNOS/OPTN database analysis. Am J Transplant.

[36] Wille KM, Harrington KF, deAndrade JA, Vishin S, Oster RA, Kaslow RA: Disparities in lung transplantation before and after introduction of the lung allocation score. J Heart Lung Transplant 2013, 32(7):684–692. 10.1016/j.healun.2013.03.005 PMC3714222 23582477

[37] Mathur AK, Schaubel DE, Gong Q, Guidinger MK, Merion RM (2010). Racial and ethnic disparities in access to liver transplantation. Liver Transpl.

[38] Malek SK, Keys BJ, Kumar S, Milford E, Tullius SG (2011). Racial and ethnic disparities in kidney transplantation. Transpl Int.

[39] Joshi S, JG J, Ciancio G (2012). Review of ethnic disparities in access to renal transplantation. Clin Transpl.

[40] Herring AA, Woolhandler S, Himmelstein DU (2008). Insurance status of U.S. organ donors and transplant recipients: the uninsured give, but rarely receive. Int J Health Serv.

[41] McWilliams JM, Meara E, Zaslavsky AM, Ayanian JZ (2007). Use of health services by previously uninsured Medicare beneficiaries. N Engl J Med.

[42] Hook JL, Lederer DJ (2012). Socioeconomic barriers to lung transplantation: balancing access and equity. Am J Respir Crit Care Med.

[43] Medicaid and CHIP: September and October 2016 Monthly Enrollment Updated December 2016 2016. https://www.medicaid.gov/medicaid/program-information/downloads/updated-october-2016-enrollment-data.pdf. Accessed February 2, 2017.

[44] Gaffney AW, Verhoef PA, Hall JB (2016). POINT: should pulmonary/ICU physicians support single-payer health-care reform? Yes. Chest.

[45] Kovesdy CP, Norris KC, Boulware LE, Lu JL, Ma JZ, Streja E, Molnar MZ, Kalantar-Zadeh K (2015). Association of Race with mortality and cardiovascular events in a large cohort of US veterans. Circulation.

[46] Freeman MA, Pleis JR, Bornemann K, Croswell E, Dew MA, Chang CH, Switzer GE, Langone A, Mittal-Henkle A, Saha S (2016). Has the Department of Veterans Affairs found a way to avoid racial disparities in the evaluation process for kidney transplantation?. Transplantation.

[47] Stephenson AL, Sykes J, Stanojevic S (2017). Survival comparison of patients with cystic fibrosis in Canada and the United States: a population-based cohort study. Ann Intern Med.

