# Supplementary material for: Disparities in pulmonary fibrosis care in the United States: an analysis from the Nationwide Inpatient Sample
Source: BMC Health Serv Res. 2018 Aug 8;18:618. doi: 10.1186/s12913-018-3407-0 (PMC6083621; doi:10.1186/s12913-018-3407-0)
Supplement: Supplementary file 1 — Diagnostic codes for other ILDs. Lists ICD-9 codes for other interstitial lung diseases. Note on disposition Includes notes on disposition variables. (DOCX 15 kb) [file 12913_2018_3407_MOESM1_ESM.docx]

**Additional file 1**

**Diagnostic codes for other ILDs**

**ICD Description**

135 Sarcoidosis

237.7 Neurofibromatosis

272.7 Lipidoses

277.3 Amyloidosis

277.8 Other specified disorders of metabolism – includes eosinophilic granuloma

446.21 Goodpasture’s syndrome

446.4 Wegener’s granulomatosis

495 Extrinsic allergic alveolitis

500 Coal workers’ pneumoconiosis

501 Asbestosis

502 Pneumoconiosis due to other silica or silicates

503 Pneumoconiosis due to other inorganic dust

504 Pneumoconiosis due to inhalation of other dust

505 Pneumoconiosis, unspecified

506.4 Chronic respiratory conditions due to fumes or vapours

508.1 Chronic and other pulmonary manifestations due to radiation

508.8 Respiratory conditions due to other specified external agents

516.0 Pulmonary alveolar proteinosis

516.1 Idiopathic pulmonary haemosiderosis

516.2 Pulmonary alveolar microlithiasis

516.8 Other specified alveolar and parietoalveolar pneumonopathies

516.9 Unspecified alveolar and parietoalveolar pneumonopathies

517 Lung involvement in conditions classified elsewhere

517.2 Lung involvement in systemic sclerosis

517.8 Lung involvement in other diseases classified elsewhere

518.3 Pulmonary eosinophilia

555 Regional enteritis

710.0 Systemic lupus erythematosus

710.1 Systemic sclerosis

710.2 Sjögren’s disease

710.3 Dermatomyositis

710.4 Polymyositis

714.81 Rheumatoid lung

720 Ankylosing spondylitis

759.5 Tuberous sclerosis

_______________________________________________

Source based on: Raghu G, Chen SY, Hou Q, Yeh WS, Collard HR. Incidence and prevalence of idiopathic pulmonary fibrosis in US adults 18-64 years old. *The European respiratory journal* 2016; **48**(1): 179-86. However, as explained in the text, ICD-9 515 (Post-inflammatory pulmonary fibrosis), which is on Raghu’s list, is excluded here.

**Note on disposition variables**

We relied on two disposition variables, “Dispub92” and “Dispub04.” “Dispub92” applies to pre-2008 discharges and follows the UB-04 claim form, while “Dispub04” applies to discharges in 2007 and onwards, and follows the UB-92 claim form. There is thus one year of overlap. Individuals with code 62 for either Dispub92 or Dispub04 were categorized as having been transferred to a rehabilitation center, while all others were categorized as not being transferred to a rehabilitation center. For Dispub 92, code 62 is defined as: “Discharge, transferred to another rehabilitation facility including rehabilitation distinct part units of a hospital, beginning in 2001 data.” For Dispub04, code 62 is defined as: “Discharged/transferred to an Inpatient Rehabilitation Facility (IRF) including Rehabilitation Distinct part unit of a hospital”).
