# Supplementary material for: Disparities in pulmonary fibrosis care in the United States: an analysis from the Nationwide Inpatient Sample
Source: BMC Health Serv Res. 2018 Aug 8;18:618. doi: 10.1186/s12913-018-3407-0 (PMC6083621; doi:10.1186/s12913-018-3407-0)
Supplement: Supplementary file 2 — Table S1. Adjusted odds of outcomes, IPF “narrow” cohort (ICD9 516.3 only). Results of the sensitivity analysis using the narrower definition of IPF (hospitalizations with ICD-9 code 516.3 only). (DOCX 19 kb) [file 12913_2018_3407_MOESM2_ESM.docx]

Additional file 2: **Table S1.** Adjusted odds of outcomes, IPF “narrow” cohort (ICD9 516.3 only)^a^

| Exposure | Lung Transplant | | Death | | Rehabilitation transfer^b^ | | VATS biopsy^b^ |  |
| --- | --- | --- | --- | --- | --- | --- | --- | --- |
|  | Odds Ratio  (95% CI) | P-Value | Odds Ratio  (95% CI) | P-Value | Odds Ratio  (95% CI) | P-Value | Odds Ratio  (95% CI) | P-Value |
| Insurance |  |  |  |  |  |  |  |  |
| Non-medicaid | *Reference* | |  |  |  |  |  |  |
| Medicaid | 0.24 (0.11, 0.52) | <0.001 | 0.99 (0.79, 1.25) | 0.94 | 1.22 (0.47, 3.17) | 0.69 | 1.04 (0.39, 2.77) | 0.95 |
| Uninsured | 0.37 (0.09, 1.57) | 0.18 | 0.94 (0.62, 1.44) | 0.78 | 1.07 (0.23, 5.01) | 0.93 | 0.51 (0.06, 4.01) | 0.52 |
|  |  |  |  |  |  |  |  |  |
| ZIP Income Quartile |  |  |  |  |  |  |  |  |
| Quartile 1 | 0.46 (0.29, 0.75) | <0.01 | 0.90 (0.78, 1.04) | 0.15 | 1.17 (0.67, 2.05) | 0.58 | 0.70 (0.33, 1.48) | 0.35 |
| Quartile 2 | 0.61 (0.44, 0.85) | <0.01 | 0.99 (0.86, 1.14) | 0.89 | 0.93 (0.54, 1.61) | 0.80 | 1.22 (0.65, 2.29) | 0.54 |
| Quartile 3 | 0.68 (0.49, 0.97) | 0.03 | 0.95 (0.83, 1.08) | 0.42 | 1.45 (0.92, 2.27) | 0.11 | 1.21 (0.66, 2.21) | 0.54 |
| Quartile 4 (Highest) | *Reference* | |  |  | *Reference* |  | *Reference* |  |

^a^ Analyses for death, rehab, and VATS were adjusted for age, race, gender, insurance, year, zip income quartile, hospital region, hospital location, hospital teaching status, hospital bedsize, and AHRQ Elixhauser Comorbidity index for in-hospital mortality. Analyses for lung transplantation adjusted for these same variables, but not hospital teaching status nor urban/rural location, because the proportion of lung transplantations exhibiting these characteristics was either 0% or 100%.

^b^ The analysis for rehabilitation transfer and VATS biopsy demonstrated questionable model fit.
